# Waiting for total knee replacement surgery: factors associated with pain, stiffness, function and quality of life

**DOI:** 10.1186/1471-2474-10-52

**Published:** 2009-05-20

**Authors:** François Desmeules, Clermont E Dionne, Étienne Belzile, Renée Bourbonnais, Pierre Frémont

**Affiliations:** 1 Population Health Research Unit (URESP), Research Centre of the Laval University Affiliated Hospital (CHA), Quebec, QC, Canada; 2 Department of Social and Preventive Medicine, Faculty of Medicine, Laval University, Quebec, QC, Canada; 3 Department of Rehabilitation, Faculty of Medicine, Laval University, Quebec, QC, Canada; 4 Laval University Hospital (CHUQ), Quebec, QC, Canada; 5 Community Health Care Centre (CSSS) de la Vieille Capitale, Quebec, QC, Canada

## Abstract

**Background:**

Recent evidences show that education and rehabilitation while waiting for knee replacement have positive effects on the patients' health status. Identification of factors associated with worse pain, function and health-related quality of life (HRQoL) while waiting for surgery could help develop pre-surgery rehabilitation interventions that target specifically these factors and prioritize patients that may benefit the most from them. The objectives of this study were to measure pain, stiffness, function and HRQoL in patients at enrolment on waiting lists for knee replacement and to identify demographic, clinical, socioeconomic and psychosocial characteristics associated with these outcomes.

**Methods:**

This study is part of a broader study measuring the effects of pre-surgery wait in patients scheduled for knee replacement. From 02/2006 to 09/2007, 197 patients newly scheduled for total knee replacement were recruited from the waiting lists of three university hospitals in Quebec City, Canada. Pain, stiffness and function were measured with the Western Ontario and McMaster Osteoarthritis Index (WOMAC) and HRQoL was measured with the SF-36 Health Survey. Stepwise multiple regression analysis was used to assess the strength of the associations between the independent variables and the WOMAC and SF-36 scores.

**Results:**

The scores of all eight HRQoL physical and mental domains of the SF-36 were significantly lower than aged matched Canadian normative data (p < 0.05). Contralateral knee pain, higher psychological distress, higher body mass index (BMI) and the use of a walking aid were significantly associated with worse function (p < 0.05) and contributed to 22% of the variance of the WOMAC function score (multiple r = 0.47). A higher BMI, the use of a walking aid, contralateral knee pain and advanced age were significantly associated with worse physical function (p < 0.05) and contributed to 17% of the variance of the SF-36 HRQoL physical functioning score (multiple r = 0.41).

**Conclusion:**

Patients waiting for knee replacement have poor function and HRQoL. Characteristics that were found to be associated with these outcomes could help develop pre-surgery rehabilitation program and prioritize patients that may benefit the most from them. Such programs could include interventions to reduce psychological distress, therapeutic exercises targeting both knees and weight loss management.

## Background

Knee replacement surgery is a common surgical procedure that allows for an effective reduction of pain and adequate restoration of function for the vast majority of patients suffering from advanced knee osteoarthritis or other forms of arthritis. [1] In the last decades, the growing needs of the population have made this procedure, along with hip replacement, the second most popular orthopaedic surgery. [2] In Canada, in 2006, the rate of knee replacements reached 106.9/100 000 persons, in sharp progression from the past decade. [3] This sharp rise in demand has translated into growing waiting lists. Governments have tried to tackle this problem, and with the allocation of new funding and the development of new policies, more patients are being operated. [4] But wait times remain a problem; recent Canadian data show that, depending on the province, the median pre-surgery wait time range from 112 to 291 days and still today an important proportion of patients are not operated within six months, the maximum acceptable waiting time benchmark established in Canada. [5,6]

Waiting for knee replacement surgery represents a significant burden for patients as they experience great pain, suffer functional limitations and loss of health-related quality of life (HRQoL) for many months. Some authors have suggested that long delays for surgery could result in patient's deterioration in terms of pain, functional limitations and HRQoL and may have negative impacts on post-surgery outcomes. [7-9] To lighten the burden of patients waiting for knee replacement, some authors have suggested that reducing wait time is not the only strategy. Some actions should target the reduction of pain and the improvement of function and HRQoL of patients while they are on waiting lists. [10] Two recent studies have shown that education and rehabilitation while waiting for total joint replacement (also called *prehabilitation*) have positive effects on the patients' health status and could lead to better outcomes postoperatively. [11,12] Until recently prehabilitation was believed to have little effect,[13] but these two studies included a full therapeutic exercises program and were carried out for a longer period of time resulting in more positive outcomes. Therefore, if prehabilitation interventions were efficacious for this population, there is a need to identify factors associated with worse pain, function and HRQoL while waiting for surgery, as it may help develop interventions that target specifically these factors and prioritize patients that may benefit the most from them. To our knowledge, only one Australian study has identified factors associated with increased pain and functional limitations or loss of HRQoL precisely at the inclusion on surgical waiting lists. This study concluded that women had worst function than men and those with lower socioeconomic status had worst HRQoL when entering waiting lists for hip or knee replacement surgery. [14] Other studies have found that factors such as advanced age, female gender, low income, low formal education, long disease duration, high body mass index (BMI), more comorbidities and high use of non-steroidal anti-inflammatory drugs (NSAIDs) were associated with worse pain, function and HRQoL in patients waiting for joint replacement. However, these factors were measured at the time of surgery or during pre-surgery waiting but not at enrolment on the pre-surgery waiting lists. [15-19]

The objectives of the current study were to measure pain, stiffness, function and HRQoL in patients at enrolment on waiting lists for total knee replacement surgery and to identify demographic, clinical, socioeconomic and psychosocial characteristics associated with these outcomes.

## Methods

### Settings

This study is part of a broader study measuring the effects of pre-surgery wait in patients scheduled for total knee replacement. From 02/2006 to 09/2007, patients were recruited from the waiting lists of the departments of orthopaedic surgery of three university hospitals in Quebec City, Canada (CHUL, HSFA and HDQ).

### Participants

Every week, patients newly enrolled on the waiting lists were contacted over the phone by a research nurse. To be eligible for the study, patients had to meet the following criteria: 1-Aged = 40 years; 2-Newly enrolled on the orthopaedic waiting lists for primary unilateral total knee replacement; 3- Residents of the province of Quebec and beneficiaries of the provincial universal health insurance coverage (Régie de l'Assurance Maladie du Québec – RAMQ); 4- Understand and speak French. Patients were excluded if they presented severe cardiac condition, degenerative disease or mental disorder. Patients with a previous contralateral knee or a hip replacement were also excluded. Those who suffered a major trauma to the knee in the previous year or were operated urgently within 30 days of being put on the waiting list were also excluded.

### Data collection

Data were collected through the review of the subjects' medical files and structured telephone interviews conducted within three weeks of the enrolment on waiting lists. The interviews lasted about 45 minutes. Daily attempts were made during weekdays and week-ends to contact new patients.

The first dependent variable was the Western Ontario and McMaster Osteoarthritis Index (WOMAC), which measures pain, stiffness and functional limitations related to the knee. [20] The 5-point Likert version was used. The WOMAC scores were transformed in order to obtain a range from 0 to 100, where a score of 100 indicated no pain, no stiffness or any functional limitations. This transformation allowed for an easier comparison with the SF-36. The WOMAC has been found to have very good reliability, convergent construct validity and responsiveness, and has been used extensively in patients suffering from knee osteoarthritis or undergoing knee replacement. [21,22]

The second dependent variable addressed HRQoL, measured with the Medical Outcomes Study 36-Item Short Form Health Survey (SF-36), a generic questionnaire on health status and HRQoL related to eight dimensions of health. [23] It allows for the calculation of two component scales: the physical component scale (PCS) and the mental component scale (MCS) as well as a specific scale for each of the eight health dimensions considered. The scores range from 0 to 100 where a score of 100 indicates optimal HRQoL. The use of the SF-36 has been extensive in population suffering from osteoarthritis[24] and in particular in patients undergoing knee replacement. [23,25-29] The reliability, validity and responsiveness of this self-administered questionnaire have been well established. [30,31]

Anthropometric data were collected through the review of the subjects' medical files after enrolment on the orthopaedic waiting lists and allowed the calculation of the BMI (in kg/m^2^). Data were typically taken from the pre-operative consultation about 6 weeks before surgery. Patients were also asked about their weight and height at the time of enrolment on the waiting list during the interview. The intraclass correlation coefficient between both measurements was very high (ICC = 0.95; 95% CI: 0.94 – 0.96).

Marital status, household living status, and clinical variables such as duration of symptoms and use of a walking aid were documented during the interview. Pain in the contralateral knee was assessed using the five questions of the WOMAC pain scale. For analyses, the score was dichotomized (presence or absence of contralateral knee pain). Using the medical files, comorbidities were documented with, the Cumulative Illness Rating Scale (CIRS). [32] This index measures the burden of chronic illness with a score that ranges from 0 to 56. This tool has been found to be reliable and valid in various settings. [33-35] Formal education, employment status, household income and social support were measured with questions drawn from the questionnaire of the 1998 Quebec Health Survey. [36] The validated and reliable social support measurement tool has three sections referring to the size of the social network, satisfaction with social life and social integration. [37] Because of time constraint, only the questions regarding the size of the social network were used. For analyses, the social support score (range: 0–150) was dichotomized around the median score. Psychological distress was established with the modified version of the Psychological Symptom Index (PSI), that measures depression and anxiety (range: 0–42). [38] Its French translation has been previously validated by Préville et al. (1992) and has been found highly reliable. [39,40]

### Analyses

Descriptive statistics were used to summarize the subjects' characteristics, WOMAC and SF-36 scores. Ninety-five percent confidence intervals (95% CI) were built around the mean WOMAC pain, stiffness and function score. Means and 95% CI of the SF-36 eight domain scores and the physical (PCS) and mental (MCS) component summary scores were compared to Canadian age-matched normative data using student t-tests.

Stepwise multiple regression analysis was used to assess the strength of the associations of the independent variables considered with the WOMAC and SF-36 scores. All three sub-scales of the WOMAC were used as dependent variables in separate models. For the SF-36, separate models were built with the physical and mental summary scores as dependent variables, as well as with the three more responsive health domain scores related to physical health: physical functioning, role-physical and bodily pain. [41] Age and gender were forced into all models. Confounding was defined as a change ≥ 10% in the regression coefficient of at least one independent variable of a model. [42] Significance levels for independent variable selection were set at 0.10 for initial model entry and at 0.05 to remain in the final model. When dependent variables showed non-normal distributions, the scores were transformed into ranks. [43] Residual plots, outliers and multicollinearity of final models were also assessed. Using simple linear regression models, assuming a type I error (α) of 0.05, power (1-β) of 0.80 and standard deviations of 25 (%) for the WOMAC score and of 45 (%) for the SF-36, sample sizes of 52 and 161 subjects would be needed to detect a 10% change in the WOMAC and the SF-36 scores, respectively. [7,28,44] A 10% change in these scores is considered clinically significant. [45,46] Statistical analyses were performed with the SAS software version 9.1 for Windows (SAS Institute Inc, Cary, NC, USA).

### Ethics

The information and consent forms were read over the phone and verbal consent was sought. The information and consent forms were then mailed to the patient and all participants signed and returned the consent form. This procedure allowed for timely data collection right at the enrolment on the orthopaedic waiting list. The study was approved annually by the Research Ethics Boards of all three hospitals.

## Results

### Participants

**Figure 1:**
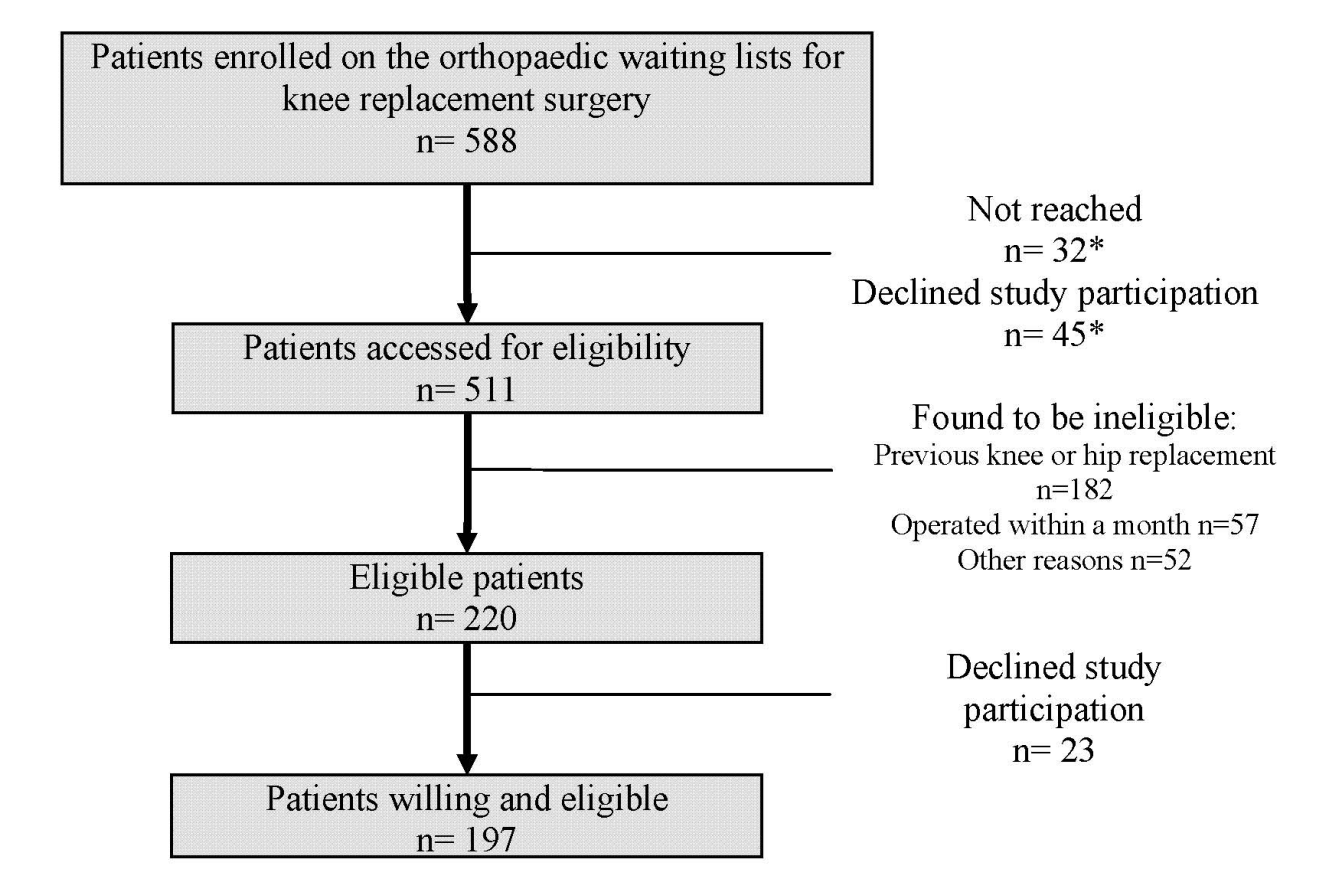
**Flowchart of patients' recruitment**. * Eligibility status unknown (considered in calculation of participation proportion).

Figure 1 presents the flow of patients considered and recruited for this study. Overall, 588 patients were enrolled on the waiting lists during the recruitment period. Thirty-two patients could not be contacted within three weeks of their inclusion on the waiting list and were excluded. Forty-five patients refused to participate before eligibility could be assessed. Once the remaining patients were assessed, 291 were found not eligible: 182 had a previous knee or hip replacement, 57 were operated within a month and 52 were excluded on other criteria. This left us with 220 eligible subjects, of whom 197 accepted to participate. The 45 patients who refused to participate before eligibility was assessed were included in the calculation of the overall eligibility proportion, (220+32)/(588-45) = 0.464 and in the calculation of the participation proportion, 197/(220 + (45 × 0.464)) = 81.8%.

### Subjects' characteristics

Table 1 presents selected characteristics of the participants. Subjects had a mean age of 67 ± 9.8 years. The majority was composed of women (64%) that were married or living in common law (63%). The mean BMI was 31.0 kg/m^2^, indicating a high frequency of obesity defined at ≥30 kg/m^2^). [47] Psychological distress was low, with a mean score of 6.9/42 when compared to the Quebec population mean (16.23 ± 14.94). [48]

**Table 1:** Characteristics of the study participants at enrolment on the waiting lists for total knee replacement (n = 197)

**Variables**	**n (%)**	**Mean (SD)**
**Demographics**		
**Age **(years)		67 (9.8)
**Gender**		
Female	126 (64)	
Male	71 (36)	
**Marital status**		
Single, separated, divorced or widowed	72 (37)	
Married or common law	125 (63)	
**Living situation**		
Living alone	45 (23)	
Not living alone	152 (77)	
**Clinical characteristics **		
BMI* (kg/m^2^)		31.0 (6.3)
Comorbidities** (/56)		6.2 (2.3)
**Duration of symptoms **(years)		8.5 (8.8)
**Contralateral knee pain**^†^		
Yes	53 (27)	
No	144 (73)	
**Use of a walking aid**		
Yes	74 (38)	
No	123 (62)	
**Socioeconomic characteristics **		
**Educational level **(part or complete)		
High school or less	112 (57)	
College or university	85 (43)	
**Employment status**		
Retired	128 (65)	
Employed	39 (20)	
Not working or sickness benefit	30 (15)	
**Household income**^‡^		
< $30 000/year	61 (36)	
≥ $30 000/year	107 (64)	
**Psychosocial characteristics**		
**Psychological distress **(/42)		6.9 (6.5)
**Social support**^§^		
Low	92 (47)	
High	105(53)	

* Body mass index

** Cumulative illness rating scale (CIRS); n = 177

^† ^Established from the WOMAC pain score for the contralateral knee

^‡ ^n = 168

^§ ^Social support was dichotomized around the median score: Low (≤ 80) and High (> 80)

### WOMAC pain, stiffness and function

Participants presented important pain, stiffness and loss of physical function as measured by the WOMAC: the mean pain score was 47.5 (95% CI: 44.9 – 50.1), the stiffness score was 42.8 (95% CI: 39.8 – 45.8) and the function score was 47.1 (95% CI: 45.1 – 49.2).

### SF-36 health-related quality of life

Summary measures of the SF-36 are presented in Table 2. Participants scored significantly lower (p < 0.05) in all eight domains of the SF-36 than the age matched adults of the general Canadian population. [49] Likewise, for the two component summary scales, the subjects' scores were significantly lower (p < 0.05) than the Canadian means.

**Table 2:** Health-related quality of life of the study participants and comparison with Canadian normative data (n = 197)

	**Mean score^**†**^(SD)**	**95% CI**	**SF-36 Canadian normative data**^**‡**^ **(Mean and SD)**	**95% CI**
**SF-36**				
Physical functioning	24.3 (17.9)*	21.8 – 26.8	75.7 (22.2)	74.9 – 76.5
Role-physical	39.3 (25.6)*	35.7 – 42.9	76.2 (36.5)	74.9 – 77.5
Bodily pain	27.5 (11.7)*	25.8 – 29.1	74.0 (23.9)	73.1 – 74.8
General health	37.1 (10.4)*	35.6 – 38.6	73.5 (18.4)	72.8 – 74.1
Vitality	39.5 (13.4)*	37.6 – 41.4	67.7 (18.1)	67.0 – 68.3
Social functioning^§^	40.9 (15.2)*	38.8 – 43.0	87.0 (19.8)	86.2 – 87.7
Role-emotional^§^	69.8 (26.3)*	66.1 – 73.5	83.4 (32.8)	82.2 – 84.6
Mental health^§^	53.4 (11.4)*	51.8 – 55.0	79.3 (15.0)	78.8 – 79.8
Physical component scale (PCS)^§^	28.2 (6.4)*	27.3 – 29.1	47.2 (9.7)	46.8 – 47.6
Mental component scale (MCS)^§^	42.9 (8.1)*	41.7 – 44.0	53.7 (8.3)	53.4 – 54.0

^† ^A higher score signs a better health-related quality of life

^‡ ^Age matched normative data

^§ ^n = 196

* Significantly lower (worse) than the Canadian normative data, p < 0.05

### Multivariate Regression Analyses

Because no important differences were found in the selection of independent variables between the initial models and the models in which the dependent variables were transformed into ranks, the final models were built with untransformed scores. Age and sex were forced into all models and no other adjustments of confounding variables were necessary.

#### WOMAC pain, stiffness and function

Results of multivariate analyses on the WOMAC scores are presented in Table 3. Contralateral knee pain, higher psychological distress and higher BMI were significantly associated with worse knee pain (p < 0.05) and explained 11% of the variance of the WOMAC pain score (multiple correlation coefficient r = 0.33). Contralateral knee pain and higher psychological distress were significantly associated with more knee stiffness (p < 0.05). Longer duration of knee symptoms was significantly associated with less knee stiffness (p = 0.003). These three subjects' characteristics explained 11% of the variance of the WOMAC stiffness score (multiple r = 0.33). Contralateral knee pain, higher psychological distress, higher BMI and use of a walking aid were significantly associated with worse knee function (p < 0.05) and explained 22% of the variance of the WOMAC function score (multiple r = 0.47).

**Table 3:** Associations between the study participants' characteristics and the WOMAC scores (n = 197)

**WOMAC SCORE**^**†**^	**β**^**‡**^	**95% CI**	***p value***
**Pain score (r^**2 **^= 0.11)**			
Contralateral knee pain	- 7.65	- 14.56 – - 3.29	0.009*
Psychological distress	- 0.45	- 0.83 – - 0.07	0.020*
BMI^§^	- 0.46	- 0.86 – - 0.05	0.026*
**Stiffness score (r^**2 **^= 0.11)**			
Contralateral knee pain	- 10.07	- 16.60 – - 3.54	0.003*
Duration of symptoms	0.53	0.18 – 0.87	0.003*
Psychological distress	- 0.55	- 1.00 – - 0.11	0.015*
**Function score (r^**2 **^= 0.22)**			
Contralateral knee pain	- 7.18	- 11.43 – - 2.93	0.001*
Psychological distress	- 0.53	- 0.82 – - 0.24	0.004*
BMI	- 0.42	- 0.72 – - 0.12	0.006*
Use of a walking aid	- 4.81	- 8.70 – - 0.94	0.015*

^† ^Stepwise multiple regression analysis. Age and gender were forced into all models.

^‡ ^Multivariate unstandardized linear regression coefficients. For each unit of the participants' characteristics, there is in average a β increase (+) or a decrease (-) on the WOMAC score. A positive β has a positive effect on the participants' condition and a negative β has a negative effect.

* p < 0.05

^§ ^BMI = Body mass index

#### SF-36 health-related quality of life

Results of multivariate analyses on the SF-36 scores are presented in Table 4. Higher BMI, use of a walking aid, contralateral knee pain and advanced age were significantly associated with worse HRQoL related to physical functioning (p < 0.05) and explained 17% of the variance of this score (multiple r = 0.41). Use of a walking aid, higher psychological distress and comorbidities were significantly associated with worse HRQoL due to physical role limitations (p < 0.05). Married subjects had significantly better HRQoL related to physical role limitations than single, separated, divorced or widowed subjects (p= 0.025). Together, these four characteristics explained 17% of the variance of the role-physical score (multiple r = 0.41). Use of a walking aid was the only characteristic associated with bodily pain and explained 5% of the variance of this score (multiple r = 0.22).

**Table 4:** Associations between the study participants' characteristics and the SF-36 health-related quality of life scores (n = 197)

**SF-36 SCORE**^**†**^	**β**^**‡**^	**95% CI**	***p value***
**Physical functioning (r^**2 **^= 0.17)**			
BMI^§^	- 0.69	- 1.10 – - 0.29	<0.001*
Use of a walking aid	- 8.28	- 13.25 – - 3.31	0.001*
Contralateral knee pain	- 6.03	- 11.37 – - 0.69	0.027*
Age	- 0.26	-0.52 – - 0.01	0.049*
**Role-physical (r^**2 **^= 0.17)**			
Use of a walking aid	- 15.23	- 22.42 – - 8.06	<0.001*
Psychological distress	- 0.71	- 1.23 – - 0.18	0.008*
Marital status (Married or common law)	8.47	1.08 – 15.85	0.025*
Comorbidities	-1.46	- 2.95 – - 0.03	0.044*
**Bodily pain (r^**2 **^= 0.05)**			
Use of a walking aid	- 3.85	- 7.27 – - 0.44	0.027*
**Physical component scale - PCS (r**^2^**= 0.08)**°			
Use of a walking aid	- 2.90	- 4.78 – - 1.02	0.003*
BMI	- 0.17	- 0.32 – - 0.02	0.030*
**Mental component scale – MCS (r^**2 **^= 0.42)**°			
Psychological distress	- 0.74	- 0.88 – - 0.60	<0.001*
Low social support	- 2.28	- 4.14 – - 0.41	0.017*
Contralateral knee pain	- 2.60	- 4.61 – - 0.59	0.011*

^† ^Stepwise multiple regression analysis. Age and gender were forced into all models.

^‡ ^Multivariate unstandardized linear regression coefficients. For each unit of the participants' characteristics there is on average a β increase (+) or a decrease (-) on the SF-36 score. A positive β has a positive effect on the participants' condition and a negative β has a negative effect.

* p < 0.05

^§ ^BMI = Body mass index

° n = 196

For the physical component scale, use of a walking aid and higher BMI were significantly associated with worse physical HRQoL and explained 8% of the variance of this score (multiple r = 0.28). Higher psychological distress, low social support and contralateral knee pain were significantly associated with worse mental HRQoL and explained 42% of the variance of the mental component score (multiple r = 0.65).

## Discussion

In this cross-sectional study, 197 patients were recruited at the time of enrolment on waiting lists for total knee replacement to measure pain, stiffness, function and HRQoL and to identify demographic, clinical, socioeconomic and psychosocial characteristics associated with these outcomes. We found that subjects reported important pain, stiffness and loss of function. HRQoL was also significantly impaired in these subjects, compared to the Canadian norms, for all the domains and components measured. These findings likely reflect the surgical indication of the subjects' condition. Interestingly however, mental aspects of HRQoL were also impaired. This finding may have implication regarding prehabilitation interventions for these patients, since it suggests that it might be beneficial to include mental health interventions to better help these patients. It is coherent with previous evidence that show that good mental health is a protective factor of functional decline in subjects suffering from knee osteoarthritis. [50]

We believe that one very interesting finding of our study is that contralateral knee pain is associated with worse pain, stiffness, function and HRQoL related to the knee scheduled for replacement surgery. Although no studies have formally identified this kind of association, from a clinical point of view, it seems logical that patients suffering from both knees have a worse condition. Only one study by Merle-Vincent (2007) has looked at that specific factor but the authors did not find a significant association. [18] Clearly, further research is needed to evaluate the effects of bilateral knee pain on patient's status and outcomes while waiting, as well as after knee replacement surgery. However, this phenomenon may have important clinical implication, as conservative treatment in patients waiting for knee replacement could realistically target both knees to maximize patients' status.

Psychological distress was low in this cohort of patients waiting for knee replacement surgery. Nonetheless, it was significantly associated with worse pain, stiffness, function and HRQoL. Other studies have outlined the important role of psychological distress on the health status of patients suffering from knee pain or undergoing knee replacement surgery. [14,17] High BMI was also significantly associated with worse pain, function and HRQoL. Other studies have found that BMI is a risk factor for the incidence and progression of knee osteoarthritis [51] and that it is also related to post-operative outcomes. [15,28] In terms of treatment, weight-loss therapy and exercise have been found to be beneficial for this population and could be an important component of a prehabilitation program. [52]

Low social support was significantly associated with worse mental HRQoL in our study. This finding is compatible with the results published by Ethgen et al. (2004), who found a significant association between social support and mental and physical aspects of HRQoL in subjects suffering from knee osteoarthritis. These authors recommended that physical health interventions should also add a social support component to improve health outcomes in these patients. [53] In our study, subjects married or living in common-law had a better HRQoL compared to single, separated, divorced or widowed subjects. Further adjustment of this regression model with social support did not change the strength of the association between marital status and the role-physical component of HRQoL. Therefore, we believe that this association is more likely to be related to the help of the spouse on coping skills than to an effect of social support. [54]

Contrary to what other studies found, sociodemographic factors were not related to pain, stiffness, function or HRQoL in our study. This may results from the fact that in this cohort of Canadian patients, access to surgery is equitable as it is not diminished in subjects of lower socioeconomic backgrounds nor that workers are fast tracked to see the surgeon. [14,16,55]

Although the association between longer duration of symptoms and knee stiffness found in our study was small, it is unclear why subjects with longer duration would show less stiffness. Maybe it results from a response shift and possible adaptation to the chronic condition of knee osteoarthritis or arthritis. [56] However this would potentially reflect also in the other sections of the WOMAC, an effect we did not observe.

Several factors were significantly associated with increased pain, stiffness, loss of function and loss of HRQoL in the study subjects. One of the strengths of our study is that many of these independent factors have a consistent effect across the scales of the WOMAC or the SF-36, which further supports the validity of our results. Other strengths include a high participation proportion (81.7%), thorough and relevant independent variables selection and no indication of selection bias (there were no significant differences between participants and eligible non participants on age and gender (data not shown). The regression models were adjusted for age and gender and further adjustments with other potential confounding factors only marginally changed the strength of the associations and were therefore not kept in the final models.

This study used a cross-sectional design and therefore caution is warranted when interpreting our results. Hypothesis related to the causality of the different independent variables on patients' health status need to be validated prospectively. Nonetheless, we believe our results provide valuable information regarding the patients' condition right at their enrollment on pre-surgery waiting list for knee replacement.

Another limitation was that the main outcome measures were self-reported and we did not include performance-based measures. The WOMAC and the SF-36 have been found to be valid instruments; still, it has been reported that performance-based measures provide distinct impressions of pain and function that complement self-reported measures. [57] Therefore, the associations or strength of associations between patients' characteristics and performance-based measures could be different from the findings of our study. Although we found a significant association between low social support and worse mental HRQoL, the social support measure only reflected the size of the social network and not the two other components of social support. Therefore associations or strength of associations with the full validated social support measurement tool could be different. It is important to point out that this study focused on patients scheduled for primary unilateral knee replacement and excluded patients undergoing a revision or with a previous contralateral knee replacement or with a hip replacement, therefore results may differ for these patients.

## Conclusion

As seen in this cohort or elsewhere in other Canadian provinces or countries, patients will be waiting for many months, often for more than six months with severe pain, loss of function and poor HRQoL. [6] We acknowledge that actions should be taken to alleviate the burden of patients by reducing wait times but with the growing needs for this surgery, allocation of more resource for surgery alone is unlikely to reach its goal. We believe that other actions are needed to improve pain, function and HRQoL of patients while they are on waiting lists. Although more research is needed to evaluate the full effects of the characteristics identified in the current study, the results of the current study could help identify subjects most in need of prehabilitation and thus may be useful to prioritize patients. Pre-operative assessment already takes place a few weeks before surgery; some of the resource used there could be diverted to meet with patients at the enrollment on the orthopedic wait lists. Patients showing a high BMI, bilateral knee pain, lower social support, poorer HRQoL mental health or high psychological distress could be identified right away and enrolled on a prehabilitation programs while they wait. Further research is needed to evaluate the effects of prehabilitation interventions targeting this population of patients or these factors specifically and could lead to a new model of care for these patients.

## Competing interests

The authors declare that they have no competing interests.

## Authors' contributions

This paper reports part of the doctoral dissertation in Epidemiology of FD, realized under the supervision of CED and the co-supervision of RB. FD participated in the design, coordination and collection of data. He performed the statistical analyses, led the interpretation of results and drafted the manuscript. CED participated in the design, coordination, interpretation of results and writing of the manuscript. EB participated in the design, coordination and writing of the manuscript. RB participated in the design and writing of the manuscript. PF participated in the collection of data and the writing of the manuscript. All authors read and approved the final version of the paper.

## Pre-publication history

The pre-publication history for this paper can be accessed here:


